# Welcome to the Journal of Market Access and Health Policy under MDPI Wings

**DOI:** 10.3390/jmahp12010002

**Published:** 2024-01-01

**Authors:** Mondher Toumi

**Affiliations:** Public Health Department, Aix-Marseille University, 13385 Marseille, France; mondher.toumi@emaud.eu

The *Journal of Market Access and Health Policy* (*JMAHP*) is the first peer-reviewed open access journal that focuses on the concept of ‘market access’ from economic, technical, scientific, sociological, anthropological, psychological, and policy perspectives. This is a unique journal dedicated to market access. *JMAHP* is the official organ of the Market Access Society (MAS).

The *Journal of Market Access and Health Policy* aims to link all components that impact ‘market access’ and provide a convergent, broader perspective in a single, specialized journal. The *Journal of Market Access and Health Policy* serves as a reference for industry, regulators, and academics on innovative research articles, discussion papers for best practices, and valuable guidelines.

*JMAHP* is in line with MAS’s vision and mission to convey transparency and consolidate knowledge around the market access research area, engaging in a forum of debate to support international dialogue, with the overall goal of being the key reference in market access around the world.

I am honoured to continue my journey of over 10 years as Editor-in-Chief of *JMAHP*. However, I could not have achieved this without the support of an outstanding editorial team, who will not only guarantee the scientific quality of the submitted articles, but also help me develop *JMAHP* to become a key resource for academics, regulators, industry, and decision makers. The journal currently has an Editorial Board of almost 30 excellent members, with whom I am delighted to work. We are eager to develop this journal together to serve as a reference for industry, regulators, and academics that, in this way, will have free and immediate access to innovative research articles, discussion papers for best practices, and valuable guidelines. All of the Editors are highly renowned colleagues representing their specific field of expertise and will work as ambassadors for *JMAHP* around the world.

The main inspiration for developing *JMAHP* was to create a single forum to provide research education, the most up-to-date information in the rapidly evolving environment of ‘market access’, and to disseminate knowledge, new policies, expert opinions, and expertise to support transparency around this field, and to share this with colleagues around the world who take an interest in market access and health policies. 

*JMAHP* features all levels of evidence-based research, guidance, policies, debates, and hands-on information for our readers.

During its 10 years of activity, *JMAHP* has made an invaluable contribution to enhancing communication and transparency in the field of market access.

Market access is the fundamental gatekeeper to patients’ access and products’ commercial success. Its complexity is hard to examine because of the multitude of rules in each different country. In addition, the healthcare market has recently seen changes in the way in which services are provided to patients: patients are more and more informed about their condition and possible treatments, mostly due to widespread use of Internet; patient associations have become more active and are able to lobby for public funding of (often costly) treatments that can heal their condition; patients need to prioritize one General Practitioner (GP) and/or primary care unit and hospital, otherwise they might miss reimbursement from the insurer; and health care professionals are obliged to follow the prescription guidelines issued by a national Health Technology Assessment (HTA) body, available therapies in their formularies, and might have less freedom to choose treatments.

Despite a large proportion of medicines becoming cannibalized by generic products, the pharmaceutical market value is continuing to grow, but at a slower pace. The continuous growth of healthcare expenditures, and more specifically, pharmaceutical expenditures, has put healthcare insurance under unbearable pressure, while providing indisputable benefit to patients.

The late 1990s and early 2000s saw a large number of cost-containment measures which failed to control the expenditure. Thus, faced with rapidly growing health care expenditure, payers have put in place an increasing number of regulations, procedures, and cost-containment measures for pharmaceutical expenses, with two goals:

- Keep expenditure within affordable limits, or as a budget voted by the government;
- Ensure the optimal usage of available resources (best health outcomes for money spent).

The COVID-19 pandemic has caused many aspects of the health market to be perceived differently. Many potential patients—which each of us was during the pandemic—began to understand how important the rapid development of procedures is for dealing with global pandemics. The pandemic exhibited the weakness of Western health care systems, the lack of pandemic readiness, the lack of supply stock, and the high vulnerability to supply chain disruption. Pharmaceutical suppliers and the governments of each country had to reach agreements quickly to develop the most efficient vaccination program possible. Governments invested massively in pharmaceutical companies into the R&D of vaccines for ensuring the first supplies. This was unprecedented. We saw leaders failing dramatically, disrupting vaccine discovery, technology, and production. The time to produce, approve, and recommend a vaccine to the first patient was shortened from several years to half a year. This was perceived as the only way to stop another, and perhaps not the last, pandemic of the 21st century.

Currently, the global economic crisis, currency crisis, debt crisis, and geopolitical instability have experienced another turning point in shaping financial expenditure on the health market. There are more and more needs that cannot be met, despite the numerous savings generated by each government to keep the economy in the best possible shape.

Therefore, over the last few years of *JMAHP*, in addition to articles related to health economics or the main issues of market access, there are more and more articles on contemporary issues of the pharmaceutical and health markets, including the coronavirus pandemic and the use of artificial intelligence or modern gene therapies. The dynamics of research and publications have been reflected in JMAHP.

Articles in *JMAHP* will be freely available worldwide via https://www.mdpi.com/journal/jmahp without any need for registration or subscription. This will ensure maximum visibility and is completely in line with our vision to promote transparency and touch base with colleagues worldwide. Being an open access journal means that all articles can reach researchers, payers, allied health care professionals, and patient groups, as well as policy makers, media, and the general public from all parts of the world.

MDPI will work to retain *JMAHP*’s indexing status with major databases such as PubMed and Scopus, and will try to achieve indexing with Web of Science as soon as the volume of content and other required criteria can be met. Material published by *JMAHP* will be distributed by EBSCO, one of the worlds’ largest content aggregators and research database providers.

To conclude, I invite the submission of articles to *JMAHP* on any of the topic areas that would contribute to the aims of the journal.

Finally, I would like to thank our team and the MDPI staff for their continuous efforts and invaluable contributions to transfer *JMAHP* to the MDPI Editorial Office. With support from the Editorial Board and MDPI, we are committed to performing timely reviews of manuscript submissions.

Together, we are writing a new page of history for *JMAHP*.

*Mondher Toumi*

Editor-in-Chief

